# Physical Properties of Nanostructured CdO Films from Alkaline Baths Containing Saccharin as Additive

**DOI:** 10.1155/2013/172052

**Published:** 2013-06-05

**Authors:** Bünyamin Şahin

**Affiliations:** Department of Physics, Faculty of Arts and Sciences, Mustafa Kemal University, 31034 Hatay, Turkey

## Abstract

Nanostructured cadmium oxide (CdO) films were fabricated on glass substrates from alkaline baths containing saccharin as an additive by a successive ionic layer adsorption and reaction (SILAR) method. The effects of saccharin concentration in the bath on the structural, morphological, and optical properties were studied by means of scanning electron microscopy (SEM), X-ray diffraction (XRD), photoluminescence, and Raman spectroscopy. The analyses showed that the surface morphologies, XRD peak intensities, Raman spectroscopy, and photoluminescence properties of CdO films changed with saccharin concentration. From the results, it can be said that morphological characteristic and optical properties of the films could be calibrated by adding various saccharin percentages in the growth bath.

## 1. Introduction

In recent years, transparent conductive oxide (TCO) layers have attracted much attention due to their high optical transmittance and low resistivity. They have been obtained by different research groups because they have potential applications in photovoltaic solar cells, phototransistors, liquid crystal displays, optical heaters, gas sensors, transparent electrodes, and other optoelectronic devices [1–3].

The optical, electrical, and morphological properties of the films could also be changed by using various additives in the growth solution. One of the methods is using some organic additives in the growth bath for solution-based synthesis. In general, additives are used in aqueous solution growth methods to control the surface morphology and the crystalline structure and to refine the grain size. The optical properties, such as the band gap energy, are quite dependent on the crystal sizes [4, 5]. There are various additives such as saccharin, citric acid, and tartaric acid. The additives are especially used to control the surface morphology and to refine the grain size [6]. Saccharin is known to be used as a strong leveling agent for the surface and a grain refiner, by decreasing of the internal stresses in the deposits [7, 8]. 

Among the metal oxide semiconductors CdO is an n-type semiconductor that crystallizes in the rock salt structure (fcc) and presents an optical band gap of about 2.2 eV [9]. CdO has high electrical conductivity and optical transmittance in the visible region of the solar spectrum. Due to these properties of CdO, it is one of the promising TCO from II to VI group of semiconductors. CdO films have been prepared so far by many different methods which are chemical bath deposition (CBD) [10], pulsed laser deposition [11], sol-gel [12], magnetron sputtering [13], metal-organic chemical vapor deposition [14], and successive ionic layer adsorption and reaction (SILAR) methods [15]. There have been practically no studies of saccharin addition process on the structural, morphological, and optical properties of nanostructured CdO films using the SILAR or other methods. In this study, for the first time, we report the effect of saccharin adding process on the structural, morphological, and optical properties of nanostructured CdO films.

The primary aim of this paper is to present the results of SEM, XRD, PL, and Raman analysis carried out for CdO films as a function of saccharin concentration. Structural properties as well as crystal quality were examined using XRD and Raman spectroscopy. Film morphology was studied with SEM micrographs. Optical properties of the films were investigated by the photoluminescence technique. 

## 2. Experimental Details

In this work, we have investigated the effect of saccharin concentration during the growth of CdO films on glass substrates by the SILAR method. All the chemical reagents used in the experiments were analytical grade purchased from Sigma-Aldrich Company and Merck KGaA. The cleaning process of the substrates (microscope glass slides) consists of three steps which are cleaning in dilute sulfuric acid solution (H_2_SO_4_ : H_2_O, 1 : 5 by volume), in acetone, and in double distilled water for 5 min, each in an ultrasonic bath [16]. Synthesis of the films was described as follows. 2.66 g cadmium acetate was weighted and mixed with 100 mL double distilled water (18.2 MΩcm) to obtain 0.1 M cadmium acetate solution. Then, the solution was stirred in a magnetic stirrer at room temperature in order to get a transparent and well-dissolved solution. After stirring, the pH value of the solution was increased to *≈*12.0 by adding ammonia (NH_3_). The solution was heated to about 85°C. The substrates were dipped into the solution and kept for 30 s. Then, they were dipped into hot water (85°C) for 30 s. This cycle was applied for 20 times. To investigate the effects of saccharin concentration to the films, five series of samples were produced. The bath for the first one was pure (i.e., contains only cadmium acetate, water and ammonia), the other baths contain 1, 3, 6, and 12% saccharin, respectively. After the film growth process, Cd(OH)_2_ films were annealed at 450°C for 1 h in a Protherm PTF 12/50/450 tube furnace air atmosphere in order to convert Cd(OH)_2_ into CdO.

## 3. Results and Discussion

### 3.1. Morphological Studies

**Figure 1:**
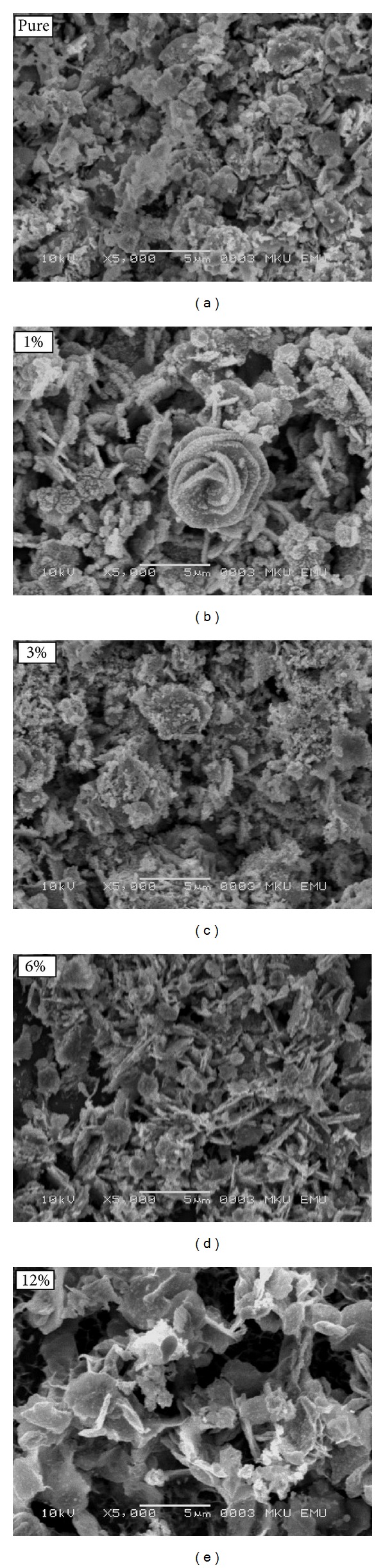
SEM images of CdO thin films prepared in the solutions having the saccharin concentrations of 0 (pure), 1, 3, 6, and 12%.

The morphology and microstructure of CdO products were examined by SEM. In order to investigate the effect of the saccharin percentage, we have conducted the experiments at five different percentages.  Figure 1 shows the structures of CdO nanoparticles which were grown in pure and saccharin added baths on the glass substrates. It can be seen that all the substrates were fully covered by CdO nanoparticles. The addition of saccharin affected the morphology on a macrovision, that is, the films seemed to be more porous with some voids. All images show homogenous and dense surfaces. The surface morphology and grain sizes of CdO films were changed considerably as a function of saccharin concentration. By using a pixels analysis program, the diameters of the grains were found as about 1730, 1390, 1510, 1471, and 1720 nm for pure and 1, 3, 6, and 12% for saccharin added samples, respectively. These SEM images show that surface morphology and grain size of the films could be controlled by saccharin percentage.

### 3.2. X-Ray Diffraction

**Figure 2:**
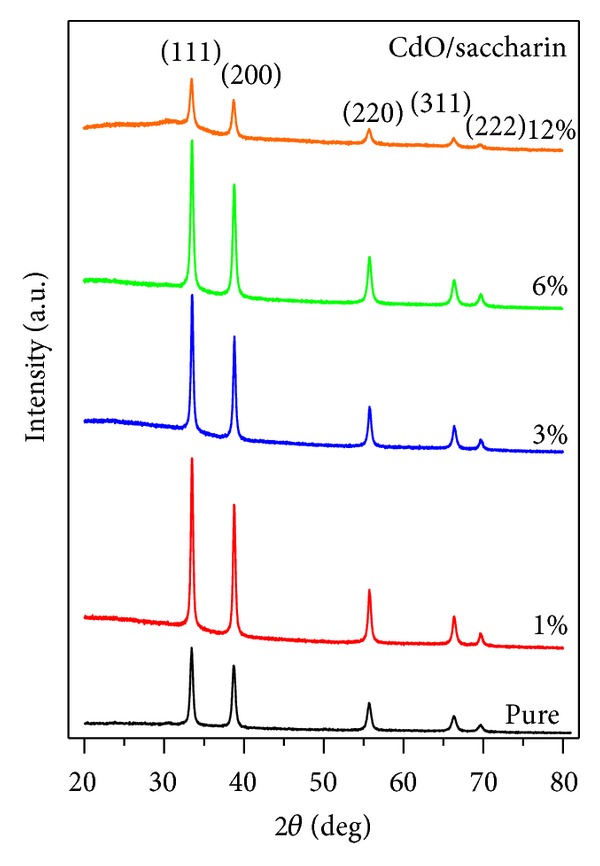
XRD patterns of CdO films as a function of saccharin concentration.

The structural properties of the pure and saccharin added CdO films were determined by XRD measurements. X-Ray diffraction analyses of the films were depicted in Figure 2. It shows typical XRD patterns of the films that have different saccharin concentration percentages in the growth solution. All diffraction peaks indicate the polycrystalline nature of CdO compound with cubic NaCl structure (JCPDS card number: 05-0640 for CdO). The intensities of (111), (200), (220), (311), and (222) and peaks in the 1, 3, and 6% saccharin added films were found to be slightly increased as seen in Figure 2 and decreased at 12% saccharin concentration. The relative peak intensities of the films are listed in Table 1. The surface morphology and grain size of CdO films were changed considerably as a function of saccharin concentration which is in agreement with the studies [7, 17–19]. Therefore, although the films' conditions are kept unchanged, the crystallinity and surface morphology change with saccharin concentration [20].

### 3.3. Photoluminescence Properties

**Figure 3:**
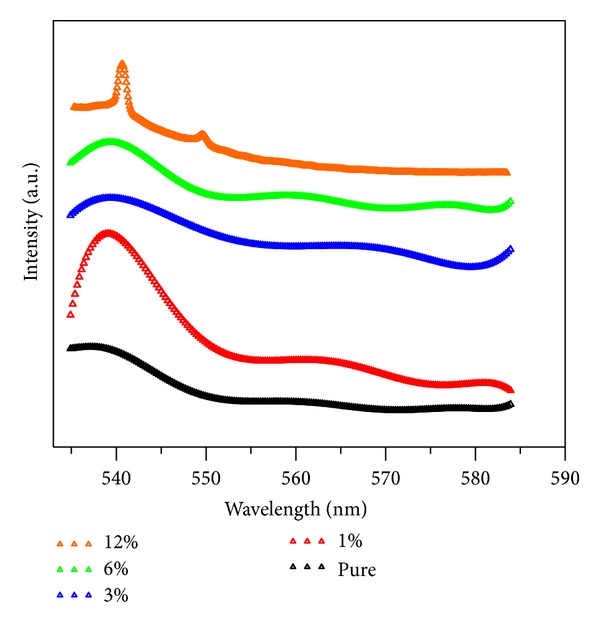
PL spectra of CdO films at room temperature as a function of saccharin concentration.

Room temperature photoluminescence spectra of CdO films measured in the visible region are shown in Figure 3. The analyses of PL spectroscopy at room temperature display various peaks. PL intensity around 540 nm which corresponds to the orange region of the electromagnetic spectrum changes with increasing saccharin concentration. The intense emission around 540, 550, and 565 nm might be attributed to the combination of the electrons from the conduction band and holes from the valence band [21]. The PL measurements show that the pure CdO film has weak luminescence behavior, but it can interact with other materials to realize its applications in luminescent devices. Actually, this fact is not valid for nanoparticles [22]. As the saccharin concentration increases, the intensity of peak emission also increases. As a result, the number of defect sites decreased with increasing saccharin concentration which is approved by the PL signals. Also, the band structure changes due to the change in the concentration of saccharin. As seen in Figure 3, the shifting in the optical band gap of CdO films can be explained by the saccharin rate which may be attributed to change in grain size [23–25]. There have been suggestions that a distribution of grain sizes may be responsible for this [26, 27].

### 3.4. Raman Properties

The assignment of Raman mode of CdO is very difficult, and it is known mostly that CdO is Raman inactive [28]. In spite of the technological importance of CdO films, the Raman properties of this material have not been further investigated. The Raman spectrum of the pure and 1, 3, 6, and 12% saccharin added CdO films in the 120–1500 cm^−1^ region was recorded by SENTERRA which is a high performance Raman microscope spectrometer using a 532 nm diode lasers. 

**Figure 4:**
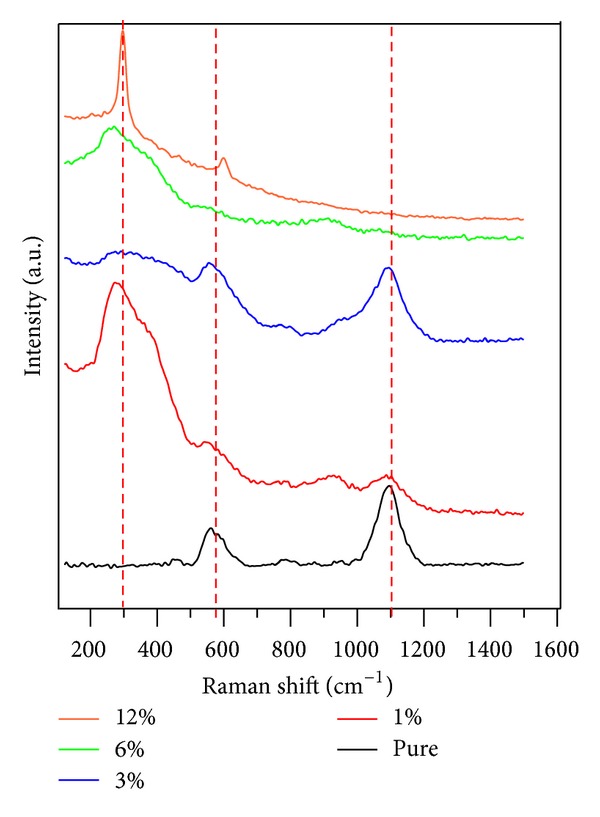
Raman spectra of CdO films at room temperature as a function of saccharin concentration.

Figure 4 shows the Raman spectra of CdO films at room temperature as a function of saccharin concentrations. CdO films revealed three distinct peaks. First sharp peak appears at 300 cm^−1^ which is not existent in pure CdO film, but when saccharin is added, it started to be observed. The peak position changed much with the saccharin concentration. The area under the Raman peak changed with the saccharin percentage and increased with higher concentration, probably due to saccharin induced surface roughness [29]. The second peak appears at 590 cm^−1^. The peak intensity and the area under the Raman peak decreased with the increase of saccharin concentration, The third peak appears at around 1100 cm^−1^. The intensity of the peak decreased with increasing saccharin concentration. As the saccharin concentration increases, the intensity of the peak around 300 cm^−1^ becames stronger, while the peak at 1100 cm^−1^ disappeared, indicating a complete phase transition. Phase transitions observed in the Raman spectra are in complete agreement with our XRD results.

## 4. Conclusion

In this study, CdO nanostructures were produced by the SILAR method. The structural, morphological, and optical properties of CdO films were investigated. The CdO films are found as polycrystalline with cubic structure. We used SEM, XRD, PL, and Raman scattering spectroscopy to determine morphology, grain size, crystallinity, and optical properties of CdO films. This may be crucial for technological device applications. The results can be concluded as follows.

- Saccharin addition affected the morphology on a macrovision. The films seem to be more porous with some voids, and all images show homogenous and dense surfaces. The surface morphology and grain sizes of CdO films were changed considerably as a function of saccharin concentration.
- Saccharin addition up to 6% to the bath causes an increase in the intensities of (111) and (200) peaks but causes a decrease at 12% concentration levels. It is observed from the SEM images and XRD patterns that the crystallite and grain sizes of the structures changed with saccharin addition.
- The PL measurement showed that the pure CdO film has weak luminescence behavior, but it can interact with other materials to realize its applications in luminescent devices.
- Saccharin added CdO films revealed three distinct Raman peaks. With the change of saccharin concentration, the intensity and the area under the Raman peaks were changed.

As a result, structural, morphological, optical, and spectroscopical characteristic, of nanostructured CdO films could be calibrated by various saccharin percentages. Thus, the nanostructured CdO materials would be hopeful for various advanced device technology. 

## Figures and Tables

**Table 1:** Peak intensities and average grain size values of CdO films as a function of saccharin concentration.

Saccharin concentration	Relative peak intensity		Average grain Size (nm)
	(111)	(200)	
0%	6937	5558	1730
1%	15460	11902	1390
3%	12983	9794	1510
6%	13784	10395	1571
12%	6500	4796	1720
